# Therapeutic Potential of Pien Tze Huang on Experimental Autoimmune Encephalomyelitis Rat

**DOI:** 10.1155/2018/2952471

**Published:** 2018-02-27

**Authors:** Xuemei Qiu, Hui Luo, Xue Liu, Qingqing Guo, Kang Zheng, Danping Fan, Jiawen Shen, Cheng Lu, Xiaojuan He, Ge Zhang, Aiping Lu

**Affiliations:** ^1^Institute of Basic Research in Clinical Medicine, China Academy of Chinese Medical Sciences, Beijing 100700, China; ^2^School of Life Science and Engineering, Southwest Jiaotong University, Chengdu 611756, China; ^3^Law Sau Fai Institute for Advancing Translational Medicine in Bone & Joint Diseases, School of Chinese Medicine, Hong Kong Baptist University, Kowloon Tong, Hong Kong

## Abstract

Multiple sclerosis (MS) is a chronic inflammatory demyelinating disease of the central nervous system (CNS). There is still lack of commercially viable treatment currently. Pien Tze Huang (PZH), a traditional Chinese medicine, has been proved to have anti-inflammatory, neuroprotective, and immunoregulatory effects. This study investigated the possible therapeutic effects of PZH on experimental autoimmune encephalomyelitis (EAE) rats, a classic animal model of MS. Male Lewis rats were immunized with myelin basic protein (MBP) peptide to establish an EAE model and then treated with three doses of PZH. Clinical symptoms, organ coefficient, histopathological features, levels of proinflammatory cytokines, and chemokines as well as MBP and Olig2 were analyzed. The results indicated that PZH ameliorated the clinical severity of EAE rats. It also remarkably reduced inflammatory cell infiltration in the CNS of EAE rats. Furthermore, the levels of IL-17A, IL-23, CCL3, and CCL5 in serum and the CNS were significantly decreased; the p-P65 and p-STAT3 levels were also downregulated in the CNS, while MBP and Olig2 in the CNS of EAE rats had a distinct improvement after PZH treatment. In addition, PZH has no obvious toxicity at the concentration of 0.486 g/kg/d. This study demonstrated that PZH could be used to treat MS.

## 1. Introduction

Multiple sclerosis (MS) is the most frequent chronic inflammatory autoimmune neurodegenerative disorder of the central nervous system (CNS) with the hallmarks of focal demyelination and inflammatory cell infiltration in the brain and the spinal cord [1]. It is a debilitating disease with high disability and recurrence rates, endangering over one million people worldwide [2]. The etiology and pathogenesis of MS are still complicated and elusive [3]. Cytokines play essential roles in mediating and regulating the inflammatory response in the CNS during MS. A key player is interleukin- (IL-) 17 which was thought to modulate neuroinflammatory and demyelinating process [4, 5]. Suppression of IL-17 signaling could alleviate EAE [6]. In addition, increasing evidence demonstrates that IL-23 induces IL-17 expression and allow the crucial role of the IL-23/IL17 pathway in MS to be recognized [7, 8]. Besides proinflammatory cytokines, some chemokines also play important roles in inflammatory process by mediating immune cells trafficking across the blood-brain barrier and modulating their transfer to lesion sites [9].

At present, the treatment of MS is limited to chemically synthesized drugs and several biological reagents, such as IFN-*β*, glatiramer acetate, and natalizumab [10], which are not always effective and are often associated with severe side effects [11]. Thus, the identification of more effective and safe agents is urgently required.

Pien Tze Huang (PZH), a well-known traditional Chinese formulation, has been widely used in various inflammatory diseases. Its main ingredients such as musk, calculus bovis (Niuhuang or ox's gallstone), Shedan (snake's gall), and *Notoginseng Radix et Rhizoma* (Tianqi or Sanqi) have been shown to exert anti-inflammatory, immunoregulatory, and neuroprotective functions [12–14]. Emerging evidences demonstrated that PZH could affect the expression of several inflammation-related factors. It showed a regulatory effect on NF-*κ*B which is closely related to the expression of inflammatory factors [15]. And it could also inhibit STAT3 signaling which plays important roles in the pathogenesis of MS/EAE [16, 17]. Moreover, recent studies showed that ginsenoside Rg1 and Rd, the main active ingredients in *Notoginseng Radix et Rhizoma*, exerted a good effect on the EAE model [18, 19].

Considering these findings, we hypothesized that PZH might be used for MS treatment. Experimental autoimmune encephalomyelitis (EAE) is the most commonly used experimental model for MS. Guinea pig myelin basic protein- (MBP-) induced EAE in rats showed severe CNS inflammation, which is usually used for the study of acute CNS inflammation [20, 21]. Therefore, in the present study, we used the EAE rat model to investigate the potential therapeutic effects of PZH on MS. An earlier version of this work was presented as an abstract at the 15th Meeting of the Consortium for Globalization of Chinese Medicine (CGCM), 2016. We further investigated the therapeutic effects and explore the action mechanism of PZH on EAE rat in the present study.

## 2. Materials and Methods

### 2.1. Animals

Male Lewis rats (8–10 weeks old) weighing between 250 g and 300 g used in this study were purchased from Beijing Vital River Laboratories (Beijing, China). They were housed in a room maintained at a 12-hour light/dark cycle (temperature 22–25°C and relative humidity 40–60%). All rats had access to food pellets and filtered water ad libitum and were given one week to adapt to the new environment. All protocols used here received approval from the Ethical Animal Care and Use Committee in China Academy of Chinese Medical Sciences and Hong Kong Baptist University.

### 2.2. Induction of EAE

An active EAE model was established following a published protocol [22]. Male Lewis rats were inoculated subcutaneously (sc) in the pad of the left hind paw with 100 *μ*L antigenic emulsion containing equal volumes of saline with 20 *μ*g of guinea pig myelin basic protein (MBP) (Sigma-Aldrich, St. Louis, MO) and complete Freund's adjuvant (CFA) (Chondrex, Redmond, WA, USA) with *Mycobacterium tuberculosis* (2 mg/mL). Daily weight was recorded, and clinical signs were evaluated, using the following 5-grade scale [23]: 0, no clinical signs; 1, limp tail; 2, hind leg weakness; 3, paraplegia and incontinence; 4, quadriplegia; and 5, moribundity or death.

### 2.3. Treatment

Pien Tze Huang (PZH) was produced by Zhangzhou Pien Tze Huang Pharmaceutical Co. Ltd. (Zhangzhou, China; FDA approval no. Z35020243). Stock solution of PZH was prepared by dissolving the PZH powder in saline, and the sample was fully blended again prior to use. Six groups were set up including the normal group, model group, prednisone acetate (PA) group (5 mg/kg/d), PZH low dose (PZH-L) group (0.054 g/kg/d), PZH middle dose (PZH-M) group (0.162 g/kg/d, equal to the clinical dose), and PZH high dose (PZH-H) group (0.486 g/kg/d). Rats for the drug groups were given daily different drugs for three weeks from day 10 (at the disease onset) after immunization, while the same volume of normal saline was given to rats for normal and model groups daily. All agents were intragastrically administered in a volume of 1 mL/100 g. After treatment, the heart, liver, spleen, lungs, and kidneys were removed and weighed for organ coefficients after washing off the blood. The sera were collected for ELISA analysis and blood biochemical determination. The whole brain and spinal cord were separated for hematoxylin-eosin (H&E) and immunohistochemical (IHC) analysis.

### 2.4. Histopathology

The brain and spinal cord were dissected after fixed in 10% neutral formalin for 48 h and embedded in paraffin after being embathed successively with different gradient ethanol and xylene. The paraffin sections of 6 *μ*m thick were obtained for H&E staining. The sections were observed with a LEICA DFC300 FX (Leica Microsystems Ltd.).

### 2.5. Immunohistochemistry

The sections (6 *μ*m thick) were dewaxed and hydrated by xylene and a graded series of alcohols after being incubated at 60°C for one hour. Heat-induced epitope retrieval was done in sodium citrate buffer. The activity of endogenous peroxidase was quenched with 3% hydrogen peroxide (H_2_O_2_). Sections were firstly incubated with antibodies against IL-17 (dilution 1 : 1500, Bioss, Beijing, China), IL-23 (dilution 1 : 1000, Bioss, Beijing, China), CCL3 (dilution 1 : 1000, Bioss, Beijing, China), CCL5 (dilution 1 : 1000, Santa Cruz Biotechnology, Dallas, Texas, USA), NF-*κ*B p65 (phospho S276) (dilution 1 : 1000, Abcam, Cambridge, UK), p-STAT3 (dilution 1 : 50, Cell Signaling Technology, Danvers, MA, USA), oligodendrocyte transcription factor (Olig2) (rabbit monoclonal antibody, dilution 1 : 200, Abcam, Cambridge, UK, 1 : 1000), or MBP (rabbit monoclonal antibody, dilution 1 : 1000, Cell Signaling Technology Inc., Danvers, MA, USA) overnight at 4°C, followed by incubation with Signal Stain® Boost IHC Detection Reagent (HRP, Rabbit) (Cell Signaling Technology, Danvers, MA, USA) according to the instructions from the manufacturers. The final color was detected using DAB Kit (ZSGB-BIO, Beijing, China) according to the manufacturer's instructions and counterstained with hematoxylin (Leagene, Beijing, China). PBS buffer was used instead of primary antibody as negative control. Images were captured at ×200 magnification by a LEICA DM6000B with a LEICA DFC300 FX (Leica Microsystems Ltd., Solms, Germany). Integral optical density (IOD) values of each image were calculated with an “Image-Pro Plus 6.0” software (Media Cybernetics, Rockville, MD, USA) [24].

### 2.6. ELISA

Commercial kits for IL-17A (eBioscience, San Diego, CA, USA), IL-23 (eBioscience, San Diego, CA, USA), CCL3 (USCN LIFE, Wuhan, China), and CCL5 (USCN LIFE, Wuhan, China) were used for measuring the concentration of cytokines in the serum of rats. The assays were performed following the manufacturer's protocol.

### 2.7. Blood Biochemical Determination

For the detection of hematological biochemical parameters, alanine aminotransferase (ALT), aspartate aminotransferase (AST), creatinine (CREA), and UREA nitrogen (UREA) were tested with blood biochemical commercial kits by a Japan's Hitachi 7160 automatic biochemical analyzer.

### 2.8. Statistical Analysis

Statistical analyses were performed by using SPSS 18.0 software. The experimental values were presented as the means ± SD. Comparisons of numerical data between the two groups were performed by Student's *t*-tests. Differences in the mean values of various groups were analyzed by using ANOVA. The *p* value < 0.05 was considered significant.

## 3. Results

### 3.1. PZH Ameliorated Clinical Symptoms of EAE Rats

**Figure 1:**
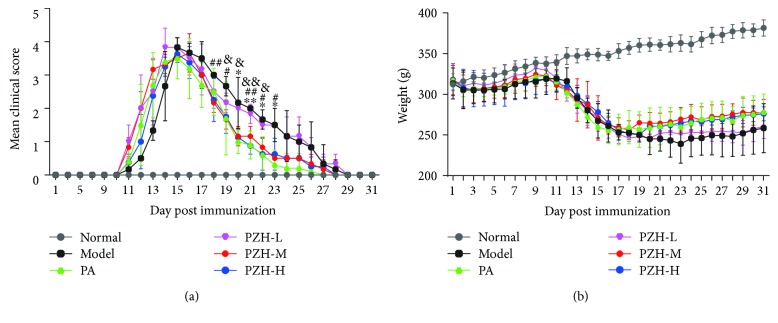
PZH ameliorated clinical symptoms of EAE rats. (a) Time course changes of the mean clinical score in rats from the respective group. (b) Time course changes of body weight in rats from the respective group. Results are shown as mean ± SD. ^∗^*P* < 0.05 and ^∗∗^*P* < 0.01; PA group versus model group. ^#^*P* < 0.05 and ^##^*P* < 0.01; PZH-M group versus model group. ^&^*P* < 0.05 and ^&&^*P* < 0.01; PZH-H group versus model group.

To examine the effect of PZH on an acute EAE model, PZH was orally administered to rats daily on day 10 post immunization. As shown in Figure 1(a), PZH effectively reduced the clinical score in remission phase, especially in the PZH-M and PZH-H groups. In addition, PZH slightly increased body weight compared with the model group, although there was no significant difference between the PZH group and the model group (Figure 1(b)). Interestingly, PZH exerted a similar effect to PA in terms of ameliorating clinical symptoms and increasing body weight, and there was no significant difference between the two groups.

### 3.2. PZH Ameliorated CNS Inflammation in EAE Rats

**Figure 2:**
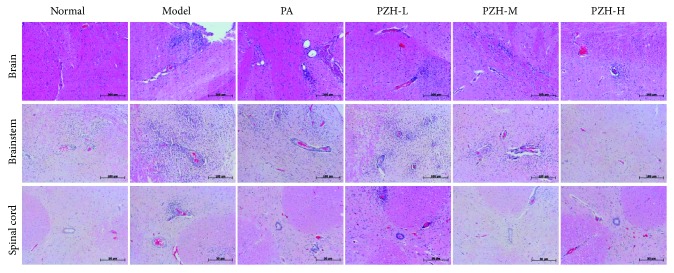
PZH ameliorated CNS inflammation in EAE rats. Rats were sacrificed at day 31, and the brain, brainstem, and spinal cord were harvested. Inflammation of the brain, brainstem, and spinal cord was analyzed by H&E staining.

To explore the anti-inflammatory effect of PZH in EAE rats, we observed the inflammation changes in different parts of the CNS including the brain, brainstem, and spinal cord by H&E staining. As shown in Figure 2, compared with the normal group, the model group showed significant vascular cuff-like changes and diffused inflammatory cell infiltration among the above three tissues. PZH treatment can dramatically reduce the degree of inflammatory lesions in the brain, brainstem, and spinal cord, which was a coincidence with the PA treatment.

### 3.3. PZH Reduced Proinflammatory Cytokine and Chemokine Production in the CNS of EAE Rats

**Figure 3:**
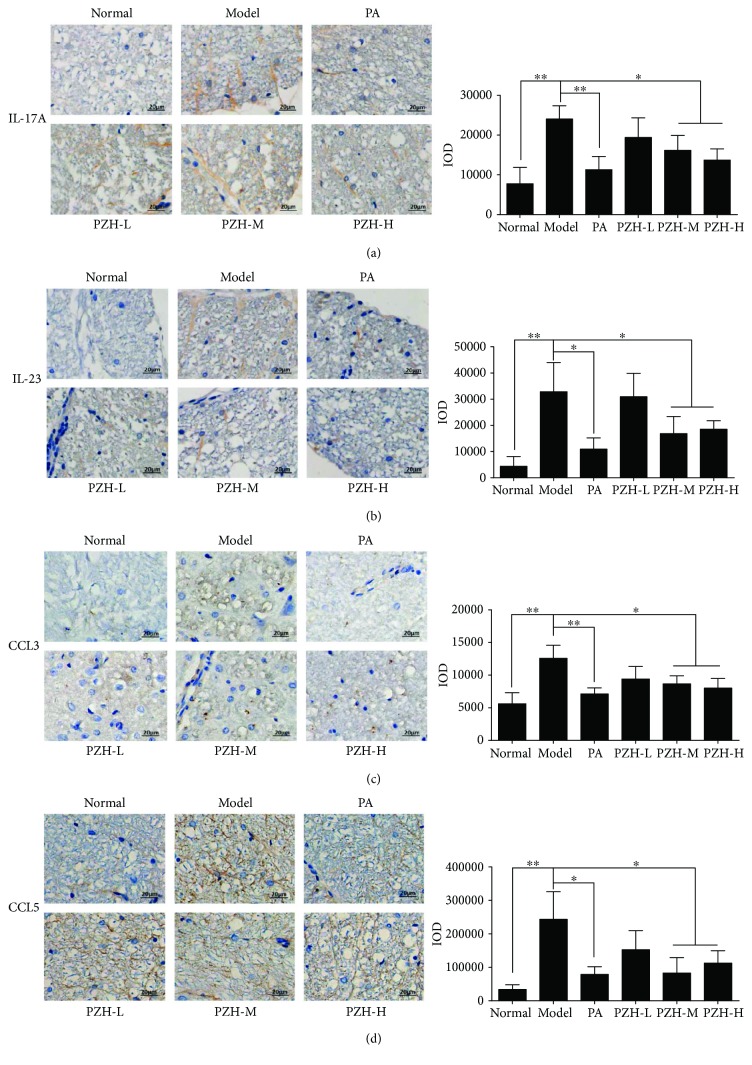
PZH decreased the levels of IL-23, IL-17A, CCL3, and CCL5 in the CNS of EAE rats. Representative immunohistochemistry images (left) and IOD means (right) of IL-23 (a), IL-17A (b), CCL3 (c), and CCL5 (d) in the spinal cord of rats from each group. Original magnification 200x. All data were shown as the mean ± SD. ^∗^*P* < 0.05 and ^∗∗^*P* < 0.01.

Proinflammatory cytokines such as IL-23/IL-17 axis and chemokines play important roles in MS inflammation progression. To further investigate the anti-inflammatory effect of PZH in EAE rats, we therefore detected IL-17A, IL-23, CCL3, and CCL5 levels in the spinal cord of EAE rats. As shown in Figure 3, compared with the normal group, the levels of IL-17A, IL-23, CCL3, and CCL5 in the spinal cord of EAE rats were remarkably increased. PZH-M and PZH-H treatment could significantly decrease the levels of these factors.

### 3.4. PZH Decreased Proinflammatory Cytokine and Chemokine Expression in Serum of EAE Rats

**Figure 4:**
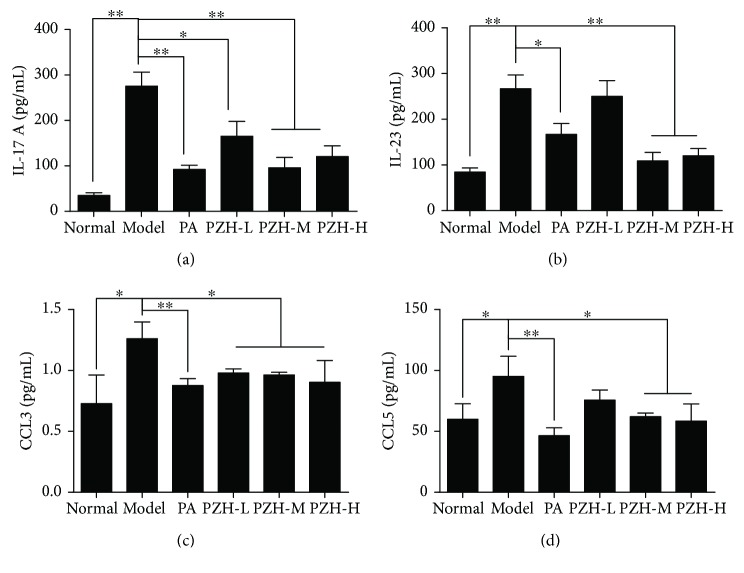
PZH reduced proinflammatory cytokine and chemokine expression in serum of EAE rats. Rats were sacrificed, and the serum was collected for ELISA analysis. The levels of IL-17A (a), IL-23 (b), CCL3 (c), and CCL5 (d) were shown, respectively. Data are shown as the mean ± SD. ^∗^*P* < 0.05 and ^∗∗^*P* < 0.01.

To observe whether the levels of IL-17A, IL-23, CCL3, and CCL5 in the serum of EAE rats had also changed, we performed ELISA analysis. As shown in Figure 4, the levels of IL-17A, IL-23, CCL3, and CCL5 were significantly increased in the model group compared with the normal group, whereas PZH could remarkably decrease these proinflammatory cytokine and chemokine levels in the serum of EAE rats, especially in the PZH-M and PZH-H groups.

### 3.5. PZH Downregulated NF-*κ*B and STAT3 in CNS of EAE Rats

**Figure 5:**
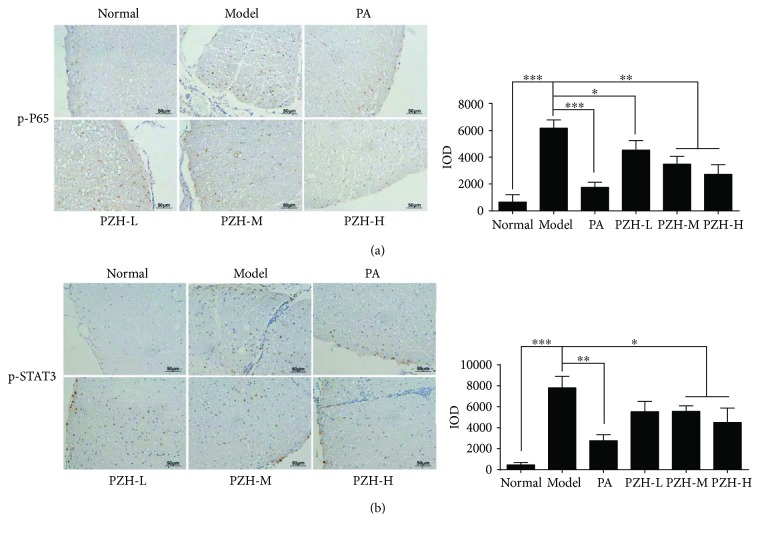
PZH downregulated the levels of p-P65 and p-STAT3 in the CNS of EAE rats. Representative immunohistochemistry images (left) and IOD means (right) of p-P65 (a) and p-STAT3 (b) in the spinal cord of rats from each group. Original magnification 200x. All data were shown as the mean ± SD. ^∗^*P* < 0.05, ^∗∗^*P* < 0.01, and ^∗∗∗^*P* < 0.001.

To further investigate the anti-inflammatory mechanism of PZH, we detected the levels of p-P65 and p-STAT3 in the spinal cord of EAE rats by immunohistochemistry. The results in Figure 5 showed that p-P65 and p-STAT3 levels were remarkably increased compared with those in normal rats. PZH treatment could significantly decrease their levels, in particular of the PZH-M and PZH-H groups.

### 3.6. PZH Increased the Expression of Olig2 and MBP in EAE Rats

**Figure 6:**
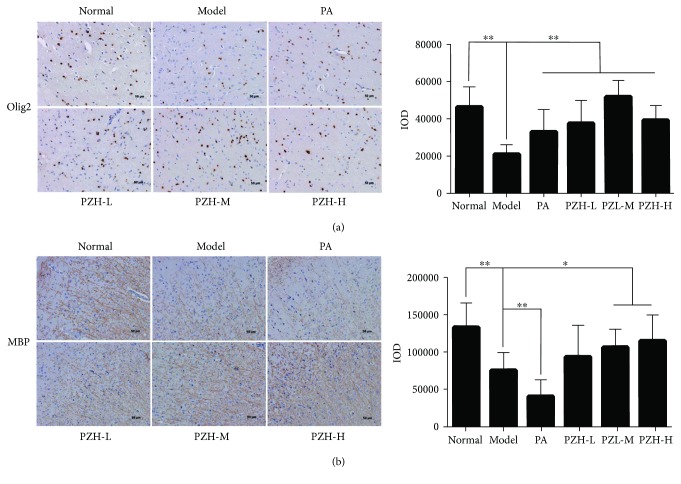
PZH increased the expression of Olig2 and MBP in EAE rats. Representative immunohistochemistry images (left) and IOD means (right) of Olig2 (a) and MBP (b) in the brain of rats from each group. Original magnification 200x. All data were shown as the mean ± SD. ^∗^*P* < 0.05 and ^∗∗^*P* < 0.01.

Because demyelination plays a crucial role in the course of disease, we wonder whether PZH has the potential in inhibiting demyelination. We then detected the levels of Olig2 and MBP in the CNS of EAE rats, two important proteins in the process of myelination of nerves. As shown in Figure 6, while the levels of Olig2 and MBP in the brain were significantly decreased in the model group compared with the normal group, PZH treatment could remarkably increase the Olig2 and MBP levels. It is worth noting that PZH may have a better effect than PA.

### 3.7. PZH Had No Significant Toxicity in EAE Rats

**Figure 7:**
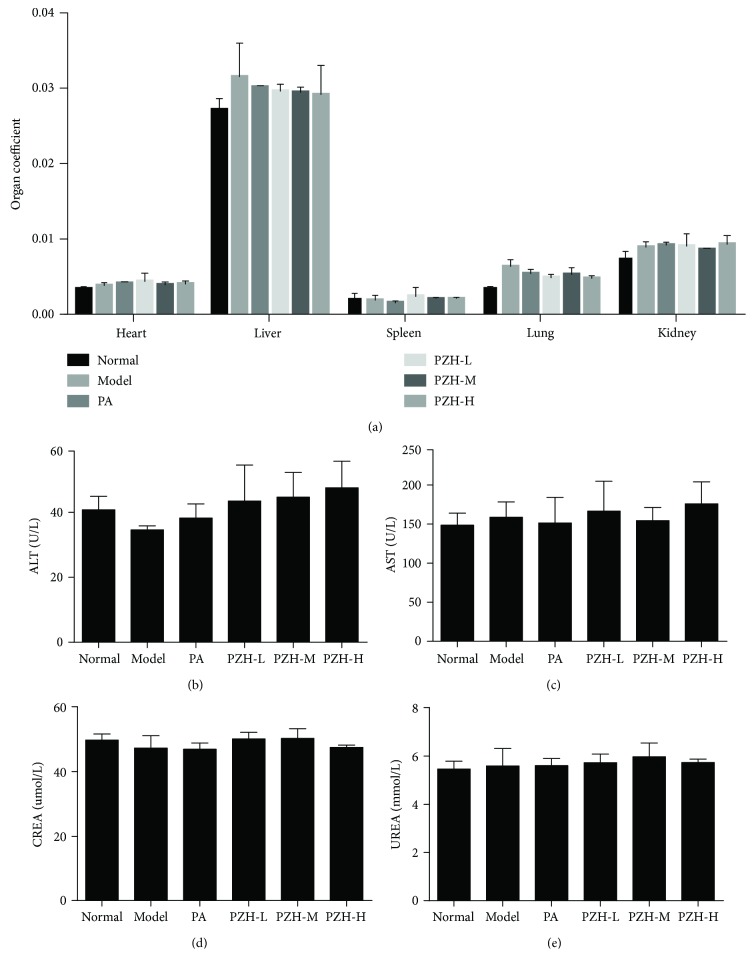
PZH has no significant toxicity in EAE rats. (a) The organ coefficient of several organs from the respective group was detected. The levels of ALT (b) and AST (c) for hepatotoxicity as well as CREA (d) and UREA (e) for nephrotoxicity were shown, respectively.

To investigate whether PZH had toxicity in EAE rats, organ coefficients as well as hepatotoxicity and nephrotoxicity were detected. As shown in Figure 7(a), organ coefficients of the heart, liver, spleen, lung, and kidney in the PZH group have no significant difference compared with the normal group. Further, as shown in Figures 7(b)–7(d), PZH treatment did not significantly change the levels of ALT and AST. Moreover, the levels of CREA and UREA in the PZH group had no obvious change either.

## 4. Discussion

MS is the prototypical inflammatory demyelinating disease of the CNS. EAE is regarded as a good model for studying MS mechanisms and developing drugs [25, 26]. It can be induced in a multitude of species and strains [20]. Rats of the inbred Lewis strain are commonly used due to its high susceptibility to EAE [27]. Lewis rats develop a monophasic EAE disease course associated with an acute onset and a spontaneous recovery, which bears resemblance to the relapse of clinical signs seen in MS. This allows the investigation of one complete episode of symptom exacerbation and remission and therefore makes this animal model a frequently utilized tool for investigating immunological and pathological characteristics of MS [28, 29]. In this study, the disease in rats became clinically evident on day 10 after immunization, and neurological signs peak on day 15 followed by complete recovery by day 28, which was consistent with previous reports [30]. According to our data, PZH effectively reduced the clinical score on remission phase in EAE rats. Moreover, it remarkably inhibited the vascular cuff-like changes and diffused inflammatory cell infiltration in the brain, brainstem, and spinal cord. Collectively, PZH exerted an obvious therapeutic effect on EAE rats.

The IL-23/IL-17 axis performs important functions in MS pathogenesis. IL-23 is predominantly secreted from activated macrophages/microglia and dendritic cells [31], driving polarization of Th17 cells, which is characterized by the production of IL-17, IL-6, and tumor necrosis factor [32]. This type of shift facilitates CNS inflammation and the development of EAE. Consistent with this, IL-23 and IL-17 in the serum and CNS have been reported to serve an important role in the pathology and immunotherapy of MS [33]. In the present study, we found that PZH effectively reduced the levels of IL-17A and IL-23 in the serum and CNS of EAE rats. Furthermore, chemokines such as CCL3 and CCL5, which are already identified to be involved in MS and EAE, are responsible for the recruitment of leukocytes to the sites of inflammation [34, 35]. In the present study, our data firstly showed that PZH significantly decreased the levels of CCL3 and CCL5 in the serum and CNS of EAE rats, suggesting that PZH may prevent inflammatory cell recruitment so as to alleviate the CNS inflammation.

In order to further explore the action mechanism of PZH remitting CNS inflammation, we detected the levels of p-STAT3 and p-P65 in the CNS of EAE rats by immunohistochemistry. As reported in previous literatures, STAT3 signaling and NF-*κ*B signaling are both proinflammatory pathways that can facilitate the expression of cytokines and chemokines [36, 37]. The severity of EAE can be alleviated by inhibiting the phosphorylation of STAT3 and/or NF-*κ*B [38, 39]. In addition, PZH was reported to suppress STAT3 signaling and NF-*κ*B signaling in other diseases [15, 16]. Similarly, we proved that PZH could also modulate these two pathways in EAE rats. To be more specific, the levels of p-STAT3 and p-P65 in the CNS of EAE rats were significantly reduced by PZH. Thus, it can be concluded that PZH inhibits the CNS inflammation in EAE rats through downregulating the STAT3 pathway and NF-*κ*B pathway.

Besides the inflammation in the CNS, demyelination is also the typical characteristics in MS. Interestingly, several studies have indicated that PZH had a neuroprotective effect [40, 41], which makes us speculate whether it also exerts the similar effect in EAE rats. Unfortunately, due to the slight demyelination in an MBP-induced rat model [20], we did not find marked remyelination in PZH-treated EAE rats (data not shown). Therefore, we further detected the changes of MBP and Olig2, two important indicators to the detection of myelin loss and regeneration, in PZH-treated EAE rats. MBP, the second most abundant protein in the central nervous system myelin, is important in the process of myelination of nerves and has already been used as the index of active demyelination [42, 43]. Olig2, restrictedly expressed in the CNS, is well known for promoting oligodendrocyte differentiation [44, 45]. Increasing evidence showed that inducible expression of Olig2 could enhance myelination and remyelination in the CNS [46]. In the present study, we indicated that PZH treatment obviously promoted the expression of MBP and Olig2 in the CNS of EAE rats, implying that PZH could facilitate the remyelination. Certainly, more EAE animal models with typical demyelination should be used to further confirm PZH's function in neural repair.

It is well known that drug safety is a key point to discover and develop new drugs, and drug safety plays a decisive role in the application of a certain drug [47]. Taking this into account, we evaluated the safety of PZH in the treatment of EAE rats. Organ coefficient is a nonspecific indicator, which can reflect the toxic effects on a target organ [48, 49]. In the present study, we found that PZH treatment did not change the organ coefficient of the heart, liver, spleen, lung, and kidney. To further confirm the safety of PZH, the levels of functional indicators of ALT and AST for the liver as well as CREA and UREA for the kidney were detected in the serum of EAE rats. Consistent with the results of organ coefficient, PZH did not show significant liver and kidney functional impairment.

In conclusion, this study demonstrated that PZH exerted a good therapeutic effect on an acute model of EAE rats, which was partly through relieving the infiltration of inflammatory cells in the CNS, suppressing the production of proinflammatory cytokines and chemokines, as well as promoting the expression of Olig2 and MBP.
